# Experiences with tailoring treatment modules in online versus face-to-face CBT

**DOI:** 10.1192/j.eurpsy.2021.74

**Published:** 2021-08-13

**Authors:** J. Boettcher, C. Wirz, S. Paskuy, M. Böttche, B. Renneberg, B. Wagner

**Affiliations:** 1 Clinical Psychology And Psychotherapy, Psychologische Hochschule, Berlin, Germany; 2 Clinical Psychology And Psychotherapy, Freie Universitaet Berlin, Berlin, Germany; 3 Clinical Psychology And Psychotherapy, Medical School Berlin, Berlin, Germany; 4 Clinical Psychological Interventions, Freie Universitaet Berlin, Berlin, Germany

**Keywords:** tailoring, internet-based, Transdiagnostic, Cognitive-Behaviour Therapy

## Abstract

Guided self-help interventions are effective in treating symptoms of various mental disorders, including depressive, anxiety, and posttraumatic stress disorders. Research also suggests that these interventions may be effective for refugee populations. However, proportion of drop-out and non-response are substantial, especially in this highly vulnerable group of patients. Tailoring treatments to the individual patient may be an important step towards improving patient-treatment fit and may help to increase success rates. While tailoring can be easily realized in face-to-face treatments, it becomes more complex in Internet-based treatments where treatment sequences are usually defined in advance. In this talk, we will present our theoretical considerations and decisions regarding the tailoring process in a randomized-controlled comparison of transdiagnostic CBT for refugee patients in an online versus face-to-face format. The trial will include N=320 Arabic speaking patients suffering from an emotional disorder. The transdiagnostic treatment includes modules for symptoms of depression, anxiety, substance abuse, post-traumatic stress, aggression, and suicidal ideation. Modules are tailored to the specific patient. We will discuss who or what should inform the tailoring decision (patient, therapist, questionnaire data, diagnostic interview) and when tailoring decisions should be made (prior and/or early and/or later in treatment). We will present options of how tailoring decisions can be standardized and be kept comparable in different treatment formats. We will present our first experiences with tailoring treatment modules to severely impaired and highly comorbid patients.

**Disclosure:**

No significant relationships.

